# Carotid atherosclerosis in people of European, South Asian and African Caribbean ethnicity in the Southall and Brent revisited study (SABRE)

**DOI:** 10.3389/fcvm.2022.1002820

**Published:** 2023-01-24

**Authors:** Rayan Anbar, Nish Chaturvedi, Sophie V. Eastwood, Therese Tillin, Alun D. Hughes

**Affiliations:** ^1^MRC Unit for Lifelong Health and Ageing, Department of Population Science and Experimental Medicine, Institute of Cardiovascular Science, University College London, London, United Kingdom; ^2^Department of Diagnostic Radiology, Faculty of Applied Medical Sciences, King Abdulaziz University, Jeddah, Saudi Arabia

**Keywords:** ethnicity, cardiovascular disease, atherosclerosis, carotid artery, medical imaging

## Abstract

**Background:**

Atherosclerotic cardiovascular disease (ASCVD) risk differs by ethnicity. In comparison with Europeans (EA) South Asian (SA) people in UK experience higher risk of coronary heart disease (CHD) and stroke, while African Caribbean people have a lower risk of CHD but a higher risk of stroke.

**Aim:**

To compare carotid atherosclerosis in EA, SA, and AC participants in the Southall and Brent Revisited (SABRE) study and establish if any differences were explained by ASCVD risk factors.

**Methods:**

Cardiovascular risk factors were measured, and carotid ultrasound was performed in 985 individuals (438 EA, 325 SA, 228 AC). Carotid artery plaques and intima-media thickness (cIMT) were measured. Associations of carotid atherosclerosis with ethnicity were investigated using generalised linear models (GLMs), with and without adjustment for non-modifiable (age, sex) and modifiable risk factors (education, diabetes, hypertension, total cholesterol, HDL-C, alcohol consumption, current smoking).

**Results:**

Prevalence of any plaque was similar in EA and SA, but lower in AC (16, 16, and 6%, respectively; *p* < 0.001). In those with plaque, total plaque area, numbers of plaques, plaque class, or greyscale median did not differ by ethnicity; adjustment for risk factors had minimal effects. cIMT was higher in AC than the other ethnic groups after adjustment for age and sex, adjustment for risk factors attenuated this difference.

**Conclusion:**

Prevalence of carotid artery atherosclerotic plaques varies by ethnicity, independent of risk factors. Lower plaque prevalence in in AC is consistent with their lower risk of CHD but not their higher risk of stroke. Higher cIMT in AC may be explained by risk factors. The similarity of plaque burden in SA and EA despite established differences in ASCVD risk casts some doubt on the utility of carotid ultrasound as a means of assessing risk across these ethnic groups.

## Introduction

Atherosclerotic cardiovascular disease (ASCVD) is the leading cause of mortality and morbidity worldwide (1). There are marked differences in ASCVD risk in different ethnic groups, even within the same country. For example the risk of coronary heart disease (CHD) is ∼1.7-fold higher in migrants from the Indian subcontinent than in people of European origin in UK (2). In contrast, people of African-Caribbean ethnicity in the UK have markedly elevated risk of stroke, but their risk of CHD is lower in comparison with Europeans or migrants from the Indian subcontinent (2). In all ethnic groups, established risk factors [e.g., blood pressure (BP), total cholesterol, high-density lipoprotein cholesterol (HDL-C), diabetes, education, alcohol consumption, and tobacco smoking] predict risk of ASCVD (3, 4), although some the prevalence of some risk factors, such as dysglycaemia, smoking and adiposity differ by ethnicity (5, 6). Previous work suggests differences in these factors only partially explain ethnic differences in ASCVD risks (2).

Detailed phenotyping of subclinical atherosclerosis may provide more insights into ethnic differences in ASCVD risk. Ultrasonography is a reliable and non-invasive technique that is widely used to assess atherosclerosis in the carotid artery (7). In addition to measurement of common carotid artery intima-media thickness (cIMT) and quantification of atherosclerotic plaques (8, 9), this method can also provide some information on plaque composition and vulnerability (10–12).

Based on the existing evidence in relation to ethnic difference in ASCVD risk, we therefore hypothesised that, in comparison with Europeans, South Asian people would have a greater burden of carotid atherosclerosis and that African Caribbean people would have similar or lower levels. We also aimed to investigate whether plaque characteristics differed by ethnicity and the potential role of established ASCVD risk factors in differences observed between ethnic groups. Individuals studied were participants in the third follow-up visit of the South and Brent Revisited (SABRE) study, a multi-ethnic longitudinal cohort that has been followed up for over 30 years.

## Materials and methods

### Participants and study design

**FIGURE 1:**
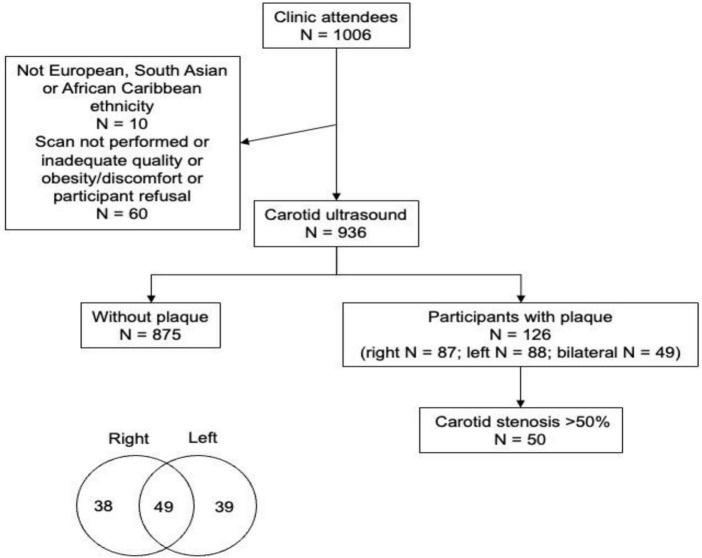
Diagram of participant flow including location of plaques as a Venn diagram.

Detailed information about the Southall and Brent Revisited (SABRE) study has been published in previously (13, 14). In brief, SABRE is a longitudinal study that recruited European (EA), South Asian (SA), and African Caribbean (AC) participants living in West and North London in 1988–1991, when they were aged 40–69 years. Participants’ ethnicity was determined by interviewers based on grand-parental origin and confirmed by participants. Surviving participants who remained in the study have undergone follow-up clinic-based investigations at 20-years (visit 2: 2008–2011) and 25-year (visit 3: 2014–2018). For the latter visit the partners of the original participants were also invited to attend. The current study included 991 individuals (437 European, 326 South Asian, 228 African Caribbean) from visit 3 (Figure 1). Ethical approval for the study was obtained from Ealing, Hounslow and Spelthorne, Parkside, and University College London Research Ethics Committees and all participants provided written informed consent.

### Clinical investigations

Participants were invited to a clinic appointment and were asked to refrain from alcohol, smoking, and caffeine for ≥12 h before attendance, and not to take their medication on the morning of the clinic visit. Information was recorded on age, sex, health behaviours, medical history, and medication (14). Height and weight were measured using a standardised protocol and body composition was measured using a Tanita BC 418 body composition analyser. Seated brachial BP was measured using an appropriately sized cuff using an automatic Omron 705 IT after 5–10 min rest according to ESH guidelines (15). The average of the second and third recordings was used as the estimate of clinic BP. Diabetes mellitus was defined according to the 1999 WHO guidelines (16), or physician diagnosis or receipt of anti-diabetes medications. Hypertension was defined as physician-diagnosed hypertension or participant-reported hypertension or receipt of BP-lowering medication. Smoking was classified into current or not. Alcohol consumption was categorised according to UK guidelines into none, ≤14 units per week or >14 units per week. Blood and urine samples were taken, and whole blood, serum, EDTA plasma and urine stored at −80°C prior to analysis. Glycosylated haemoglobin (HbA1c) was measured on an automated platform (c311, Roche Diagnostics, Burgess Hill, UK), serum total cholesterol, HDL-C and triglycerides were measured using enzymatic methods (Roche/Hitachi cobas c system). Low density lipoprotein cholesterol (LDL-C), All assays used the manufacturers calibration and quality control material.

### Ultrasound measurements

Ultrasound scans were performed by an experienced sonographer using a GE Vivid I Ultrasound system equipped with a 6-13 mHz broadband linear array transducer (12L-RS). The common carotid artery (CCA), internal carotid artery (ICA), external carotid artery (ECA) was assessed along the long- and short-axes bilaterally. Two-dimensional-images, spectral-Doppler imaging, Colour and Power Doppler were also recorded. Adequate quality of ECG signals and ultrasound images was ensured throughout the examination. A cine loop of at least five cardiac cycles at three angles (lateral, posterior, and anterior) as well as one 8-bit greyscale image captured at the R wave for each angle were acquired. cIMT and carotid lumen diameter was measured from the best visualised image over a 10 mm segment in the CCA according to the American Society of Echocardiography Carotid Intima-Media Thickness Task Force Consensus Statement (17). Plaque was defined according to the Mannheim consensus (8, 9), as a focal lesion that encroached into the carotid artery lumen by ≥0.5 mm or ≥50% of the surrounding cIMT value or had a thickness >1.5 mm as measured from the media-adventitia interface to the intima-lumen boundary. Carotid stenosis >50% was assessed by visual inspection of the B-mode ultrasound scan, using Colour and Power Doppler imaging as needed, and quantified according to NASCET criteria (18). All quantitative analyses were performed offline using validated software (AMS II) (19) that included automated measurement of plaque area, categorisation of plaque based on the Grey-Weale score (10), plaque size and estimation of grey-scale median (GSM) (20, 21). Repeatability and reproducibility of cIMT and plaque characteristics have been reported previously (22).

### Statistical analysis

Statistical analyses were performed with Stata v.17.1 (StataCorp, College Station, TX, USA). Continuous data for the sample were summarised as means and standard deviations (SD) or median (interquartile range) for skewed data, categorical data as counts and percentages. Normality was assessed through frequency histograms, QQ plots and Shapiro-Wilk tests. Comparisons between ethnic groups were made using generalized linear modelling (GLM). Two models were used to provide further insight into ethnic differences: (Model 1) non-modifiable risk factors (age and sex); (Model 2) Model 1 plus established modifiable risk factors (diabetes, hypertension, total cholesterol, HDL-C, years of education, alcohol consumption, current smoking, statin medication). Choice of covariates was based on *a priori* knowledge (4, 23). Additional sensitivity analyses were performed where diabetes was replaced by HbA1c, or where systolic BP or body mass index (BMI) (or waist hip ratio) were added to models (these models showed negligible differences from the original models and the results are not presented). The possibility of effect modification by sex was looked for in all models by including a sex × ethnicity interaction term, if this was not statistically significant both sexes were pooled for analysis, otherwise it was planned that results for both sexes would be analysed separately.

Dichotomous variables (e.g., presence of plaque or presence of carotid stenosis >50%) were modelled using GLM with a binomial and log family and link function. Ordered categorical variables with fewer than six categories [median plaque grade (manual and automatic)] were analysed using ordered logistic regression and the proportional-odds assumption was tested using an approximate likelihood ratio test. If the proportional-odds assumption was not met data were fit with partial proportional odds models using generalised ordinal logistic regression (gologit2) (24). Numbers of plaques were modelled using negative binomial models as data were expected to be over-dispersed (this was confirmed using the likelihood ratio test for alpha = 0). Risk ratios, or marginal probabilities and 95% confidence intervals (CI) were estimated from these models. Multiple linear regression models were used for continuous measures (total area of plaques, lowest GSM of all plaques, cIMT) and marginal means and CI estimated. If regression models showed evidence of heteroskedasticity, robust standard errors were calculated. Assumptions of linearity were checked by examination of residuals and if necessary, it was planned that non-linear models would be constructed using fractional polynomials. The primary analysis was a complete case analysis which is valid under the assumption that missingness was independent of outcomes. As a sensitivity analysis, models using full information maximum likelihood which is valid under the missing at random (MAR) assumption were also examined for linear models. Inference was based on a combination of *p*-values, effect sizes and CI, no adjustment was made for multiple comparisons.

## Results

Table 1 shows the characteristics of the sample stratified by ethnicity. Participants were aged between 40 and 69 years and comprised 437 EA (mean age 74 years, 62% male), 326 SA (mean age 73.2 ± 6.3 years, 59.3% male), and 228 AC (mean age 71 years, 35.6% male). On average SA were slightly younger than EA and AC were younger than both EA and SA, and there were more women in the AC sample. AC and SA people were shorter, had higher systolic BP and more diabetes and hypertension than EA. Compared with EA, SA had a higher prevalence of known CHD, more years of education, lower heart rate, lower BMI, were shorter and were less likely to be current smokers, and less likely to consume high quantities of alcohol, while AC had a lower prevalence of CHD, higher BMI, higher diastolic BP, more diabetes and hypertension and were less likely to consume high quantities of alcohol.

**TABLE 1:** Characteristics of sample by ethnicity.

Variables	Ethnicity									ANOVA or Chi^2^ *p*-values
	EA			SA			AC			
	*N*	Mean/%	(SD)	N	Mean/%	(SD)	N	Mean/%	(SD)	
Age, years	437	74.4	(6.10)	326	73.20*	(6.3)	228	70.5*	(7.91)	<0.001
Male sex	274	49.3%		194	59.3%		85	37.0%*		<0.001
Systolic blood pressure, mmHg	437	138.67	(18.3)	325	143.63*	(18.62)	228	142.26*	(16.68)	<0.001
Diastolic blood pressure, mmHg	437	78.84	(10.8)	325	78.48	(10.6)	228	81.73*^†^	(10.25)	<0.001
Heart rate, bpm	437	68.18	(11.33)	325	65.76*	(10.24)	228	68.12^†^	(11.35)	0.001
Height, cm	437	167.99	(8.7)	326	162.28*	(8.86)	228	164.12*^†^	(7.79)	<0.001
BMI, kg/m^2^	437	28.01	(4.53)	326	26.42*	(3.91)	228	29.94*^†^	(5.30)	<0.001
HbA1c, mmol/mol	423	38.61	(8.0)	314	43.27*	(9.37)	220	39.98^†^	(11.04)	<0.001
Years of education	377	11.9	(3.53)	223	13.5*	(3.77)	153	12.01^†^	(3.69)	<0.001
Diabetes mellitus	53	13%		88	28%*		54	27%*		<0.001
CHD	53	13%		59	19%*		13	6%*^†^		<0.001
Stroke	8	2%		3	1%		4	2%		0.558
Hypertension	209	49%		210	66%*		152	69%*		<0.001
Alcohol consumption										<0.001
None	107	24%		170	52%		99	43%		
≤14 units per week	262	60%		150	46%		129	56%		
>14 units per week	71	16%		7	3%		2	1%		
Current smoker	16	4%		3	1%*		8	3%^†^		0.051
Statin use	210	48%		209	64%*		85	37%*^†^		<0.001
Presence of carotid plaque(s)	74	17%		55	17%		14	6%*^†^		<0.001
Carotid stenosis >50%	22	5%		23	7%		5	2.2%^†^		0.025
cIMT, mm	407	0.89	(0.20)	311	0.89	(0.23)	212	0.91	(0.20)	0.73
Total plaque area per individual with plaque, mm^2^	74	34.4	(29.2)	55	33.6	(27.5)	14	29.4	(27.6)	0.84
Number of plaques per individual with plaque	74	1.5	(1.1)	55	1.5	(0.8)	14	1.1	(0.86)	0.35
Minimum GSM of all plaques	63	86.9	(35.5)	52	80.1	(28.8)	10	79.8	(12.7)	0.48
Class (manual) of largest plaque	55	2.84	(0.46)	46	2.87	(0.40)	7	2.86	(0.38)	0.93
Class (auto) of largest plaque	63	2.32	(0.53)	52	2.19	(0.40)	10	2.3	(0.48)	0.37

BMI, body mass index; CHD, coronary heart disease; cIMT, carotid artery intima-media thickness; GSM, greyscale median; HbA1c, glycated haemoglobin; SD standard deviation. *p*-values were calculated using Chi^2^ tests and logistic regression for categorical variable and ANOVA for continuous variables, Wald tests were used for individual comparisons. *p < 0.05 compared with Europeans, ^†^*p* < 0.05 compared with South Asians.

**FIGURE 2:**
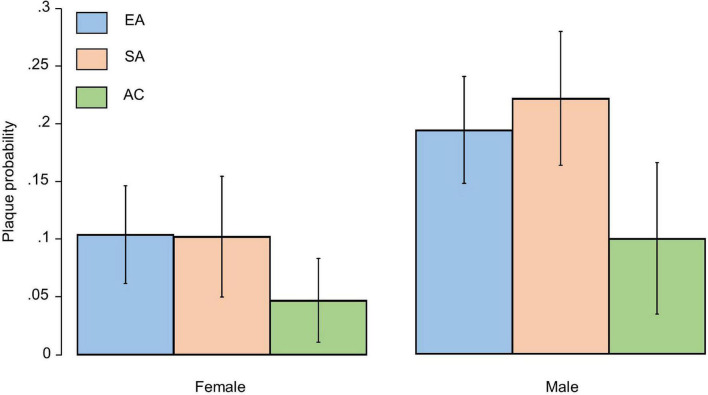
Bar plot showing marginal probabilities with 95% confidence intervals (adjusted for age) of having a plaque by sex and ethnicity. EA, European; SA, South Asian, and AC, African Caribbean.

Carotid artery intima-media thickness was similar by ethnicity in an unadjusted model, but plaques were more frequent in EA and SA than AC (Table 1); however, there was no difference between EA and SA (Table 1). Plaques were more common in men than women but there was no evidence that sex modified the ethnic differences in plaque prevalence (Figure 2). There were no marked differences in distribution of plaques by ethnicity: 57.5% of Europeans had plaques in the left carotid artery, 47.6% in the right carotid artery and 44.1% had plaques bilaterally. A total of 42.5% of South Asians had plaques in the left carotid artery, 44.1% in the right carotid artery and 32.7% had plaques bilaterally. A total of 10% of African Caribbean’s had plaques in left of carotid artery, 8.1% in the right carotid artery and 23.0% had plaques bilaterally (Figure 1). In comparison with EA, the risk ratios for having any plaque in SA after adjustment for non-modifiable, or non-modifiable plus modifiable risk factors were 1.08 (0.79, 1.46); *p* = 0.65 and 0.98 (0.66, 1.41); *p* = 0.89, respectively. For AC the comparable risk ratios were 0.48 (0.28, 0.82); *p* = 0.013 and 0.53 (0.268, 1.047); *p* = 0.068. The marginal probabilities of having one or more plaques in each ethnic group are shown in Table 2 with and without adjustment. The probability of having one or more plaques was similar in EA and SA but was lower by ∼50% in AC. Statistical adjustment had little effect on these estimates, although the estimated CI of the fully adjusted model were wider, probably as a result of the reduced sample size of the complete case analysis for non-modifiable plus modifiable risk factors (*n* = 727). Compared with EA, the risk ratio in SA for having a stenosis >50% was 1.56 (0.88, 2.76); *p* = 0.12 and 1.70 (0.81, 3.52); *p* = 0.15. For AC the risk ratios were 0.61 (0.23, 1.61); *p* = 0.32 and 0.65 (0.25, 1.70); *p* = 0.38. The limited number of stenoses in the sample (*n* = 50) made these estimates very imprecise and scope for inference was limited.

**TABLE 2:** Marginal probabilities of having any plaques by ethnicity with and without adjustment for risk factors.

Prevalence of plaque by ethnicity			*p*-value (individual comparisons)			*p*-value LR test
EA	SA	AC	EA vs. SA	EA vs. AC	SA vs. AC	
**Unadjusted**						
0.16 (0.13, 0.20)	0.16 (0.12, 0.20)	0.06 (0.03, 0.09)	0.992	<0.001	<0.001	<0.001
**Model 1**						
0.15 (0.12, 0.18)	0.16 (0.12, 0.20)	0.07 (0.03, 0.11)	0.626	0.008	0.004	0.006
**Model 2**						
0.16 (0.12, 0.19)	0.19 (0.14, 0.24)	0.08 (0.03, 0.13)	0.241	0.068	0.014	0.036

Data are marginal probabilities (95% confidence intervals). Model 1: age, sex adjusted, Model 2: Model 1 + years of education, diabetes, total cholesterol, HDL, alcohol consumption, current smoking, hypertension, statin use. LR, likelihood-ratio.

After adjustment for age and sex cIMT was higher in AC than EA or SA but there was no difference in cIMT between EA and SA (Table 3). Further adjustment for risk factors attenuated differences by ethnicity (Table 4).

**TABLE 3:** Ethnic differences in cIMT and plaque characteristics with adjustment for age and sex (Model 1).

Variables	Ethnicity			*p*-value (individual comparisons)			*p*-value LR test
	EA	SA	AC	EA vs. SA	EA vs. AC	SA vs. AC	
cIMT, mm	0.88 (0.86, 0.90)	0.89 (0.87, 0.92)	0.93 (0.90, 0.96)	0.285	0.002	0.033	0.003
Total plaque area per individual with plaque, mm^2^	40.6 (33.4, 46.6)	35.2 (27.9, 42.5)	44.5 (27.5, 61.4)	0.343	0.624	0.323	0.473
Number of plaques per individual with plaque	1.76 (1.56, 1.96)	1.64 (1.41, 1.86)	1.62 (1.10, 2.13)	0.416	0.604	0.945	0.667
Minimum GSM of all plaques	87.44 (79.60, 95.29)	79.57 (70.89, 88.25)	78.58 (58.64, 98.52)	0.187	0.414	0.929	0.350
Category of largest plaque				0.507	0.820	0.580	0.741
Class 1	0	0	0				
Class 2	0.20 (0.10, 0.30)	0.15 (0.06, 0.25)	0.23 (−0.08, 0.55)				
Class 3	0.78 (0.68, 0.87)	0.82 (0.73, 0.90)	0.75 (0.46, 1.03)				
Class 4	0.02 (−0.01, 0.05)	0.03 (−0.01, 0.07)	0.02 (−0.02, 0.06)				
Size class of largest plaque				0.121	0.849	0.296	0.247
Mild	0.01 (−0.01, 0.03)	0.02 (−0.01, 0.05)	0.01 (−0.01, 0.03)				
Moderate	0.66 (0.54, 0.77)	0.77 (0.67, 0.87)	0.63 (0.34, 0.92)				
Severe	0.33 (0.22, 0.45)	0.21 (0.10, 0.31)	0.36 (0.06, 0.67)				

All data are marginal means (95% confidence intervals) or probabilities (95% confidence intervals). BMI, body mass index; CHD, coronary heart disease; cIMT, carotid artery intima-media thickness; GSM, greyscale median; HbA1c, glycated haemoglobin; SD standard deviation; LR, likelihood-ratio.

**TABLE 4:** Ethnic differences in cIMT and plaque characteristics with adjustment for non-modifiable and modifiable risk factors (Model 2).

Variables	Ethnicity			*p*-value (individual comparisons)			*p*-value LR test
	EA	SA	AC	EA vs. SA	EA vs. AC	SA vs. AC	
cIMT, mm	0.88 (0.86, 0.90)	0.89 (0.86, 0.92)	0.92 (0.88, 0.95)	0.700	0.091	0.188	0.220
Total plaque area per individual with plaque, mm^2^	41.3 (33.9, 48.7)	34.2 (26.6, 43.1)	44.5 (26.5, 62.4)	0.285	0.742	0.332	0.402
Number of plaques per individual with plaque	1.85 (1.63, 2.07)	1.56 (1.32, 1.81)	1.57 (1.03, 2.10)	0.111	0.332	0.996	0.196
Minimum GSM of all plaques	87.41 (79.00, 95.83)	78.62 (69.23, 88.02)	75.80 (55.36, 96.23)	0.204	0.306	0.805	0.300
Category of largest plaque				0.335	0.852	0.747	0.616
Class 1	0	0	0				
Class 2	0.23 (0.11, 0.35)	0.15 (0.04, 0.25)	0.20 (−0.13, 0.52)				
Class 3	0.75 (0.64, 0.86)	0.82 (0.72, 0.92)	0.78 (0.49, 1.06)				
Class 4	0.02 (−0.01, 0.05)	0.03 (−0.02, 0.08)	0.02 (−0.03, 0.07)				
Size class of largest plaque				0.498	0.884	0.607	0.748
Mild	0.02 (−0.01, 0.04)	0.02 (−0.01, 0.06)	0.01 (−0.02, 0.05)				
Moderate	0.68 (0.56, 0.79)	0.74 (0.61, 0.87)	0.65 (0.30, 1.00)				
Severe	0.31 (0.19, 0.43)	0.24 (0.10, 0.38)	0.34 (−0.04, 0.71)				

All data are marginal means (95% confidence intervals) or probabilities (95% confidence intervals). BMI, body mass index; CHD, coronary heart disease; cIMT, carotid artery intima-media thickness; GSM, greyscale median; HbA1c, glycated haemoglobin; SD standard deviation; LR, likelihood-ratio.

In individuals with plaque, a comparison of plaque area, average number of plaques, minimum greyscale median, and plaque class is shown in Tables 3, 4 following adjustment for risk factors. Total plaque area was similar in all ethnic groups, as was plaque class and echogenicity as assessed by GSM.

## Discussion

We found ethnic differences in the prevalence of carotid plaque in a population-based sample of people in UK. People of AC ethnicity had a lower occurrence of carotid plaque than the other ethnic groups, while the burden of plaque in EA and SA was similar. The lack of difference between EA and SA was surprising considering the large excess of cardiovascular disease reported in SA and our data for carotid plaque prevalence are not consistent with previous estimates of excess CHD risk in SA (2). In those with plaque, plaque characteristics differed little between ethnic groups, in particular there was no evidence of SA having evidence of more lipid-rich or vulnerable plaques. Ethnic differences in plaque prevalence were unexplained by disparities in ASCVD risk factors. It therefore remains unclear why the AC group had a lower prevalence of carotid plaques than the other ethnic groups, however, this observation is consistent with previous work, including in SABRE, showing lower risk of CHD in people of AC ethnicity in UK, which was unexplained by conventional ASCVD risk factors (25, 26). cIMT also differed by ethnicity, after adjustment for age and sex, cIMT was higher in AC compared with the other ethnic groups, which could be consistent with their higher risk of stroke, but is inconsistent with their lower risk of CHD; this difference was attenuated after adjustment for non-modifiable and modifiable risk factors and is likely to be attributable to differences in ASCVD risk factors.

Better understanding and assessment of the prevalence of atherosclerosis and its relationship to cardiovascular risk factors in different ethnic groups is important. Such relationships may also provide insights into the pathogenesis of atherosclerosis in all ethnic groups. Our failure to identify factors explaining ethnic differences in carotid atherosclerotic plaque despite adjustment for ASCVD risk factors suggests that important determinants of ethnic differences in atherosclerosis susceptibility remain to be identified. Mechanisms related to population migration (27), socio-economic disadvantage (28) and racism (28, 29) seem plausible explanations, but given the differences observed between minority ethnic groups in this study this question merits further study. We cannot exclude genetic differences between populations of difference ancestry but currently there is little or no evidence to suggest that genetics makes a major contribution to ethnic differences in susceptibility to ASCVD (30, 31).

Previous studies have examined ethnic differences in carotid atherosclerosis, although few have included SA people. A UK community-based study found higher cIMT and lower prevalence of plaque in AC compared with EA (32) and this difference remained after adjustment for conventional ASCVD risk factors. Another UK-based study observed marginally higher cIMT in EA compared with SA despite higher prevalence of ASCVD in SA (33). In the US, the Multi-Ethnic Study of Atherosclerosis found that cIMT was higher in people of African American ethnicity, but the risk of new plaque formation was lower in African American, Hispanic and Chinese ethnicities compared with White Americans after adjustment for traditional ASCVD risk factors (34). The Diabetes Heart Study also found that African American people with T2DM had higher cIMT but lower prevalence of carotid plaque compared with those of European ancestry (35). In contrast, the Northern Manhattan Stroke study found similar maximum internal carotid artery plaque thickness (MICPT) in stroke-free African- and European- ethnicity individuals but lower MICPT in people of Hispanic ethnicity (36). A recent individual participant meta-analysis that compared the association of ASCVD risk factors with cIMT in different ethnicities from a range of countries, reported that high cIMT levels was highest amongst African American populations, similar in Asian, White and Hispanic people and lowest in African populations. In keeping with our findings, adjustment for risk factors only marginally attenuated these differences (37). Overall, despite some inconsistencies the results of these previous studies appear broadly consistent with our findings.

As has been observed in some previous studies (32–34), cIMT corresponded poorly with known risk differentials for ASCVD, especially CHD, in the ethnic groups. Plaque prevalence was consistent with the known lower risk of CHD in AC, but not with the elevated risk of ASCVD in SA or the elevated risk of stroke in AC (38, 39). This raises questions about the reliability of cIMT and plaque as a screening tool for early detection of atherosclerosis *across* different ethnic groups. For cIMT it has previously been suggested that arterial wall remodelling in response to haemodynamic stresses might complicate interpretation (40), but it is not obvious that this could explain the ethnic discordance between ASCVD risk and plaque prevalence, given the latter is generally considered a better predictor of ASCVD risk (41).

This study has limitations and strengths: it is cross-sectional so causal conclusions cannot be made. Participants were drawn from a randomly selected population-based cohort but possible bias due to non-participation, attrition, missing data and residual confounding by unmeasured or imprecisely measured variables cannot be excluded. As might be expected in a population-based sample, the frequency of carotid plaque was quite low, particularly in AC, which may have limited our ability to detect small differences in plaque prevalence or characteristics, nevertheless the precision of the estimates was sufficient to exclude disparities in plaque prevalence consistent with CVD risk differentials in South Asians. Our categorisation of ethnicity is crude and may obscure important differences within ethnic groups; (42), however, our categories reflect the original study design and correspond to the broad ethnic groups in used by the UK classification scheme (43). AC participants mostly migrated between 1950 and 1960 (i.e., around the ages of 20 to 30), while most of the SA participants arrived in the UK in the 1970’s (i.e., around 40 years old) and limited data was available about exposures, including childhood exposures and healthcare provision, that occurred prior to migration or extent of acculturation after migration. We included a comprehensive set of risk factors for ASCVD, but we acknowledge that including these risk factors, which potentially act as mediators of ethnic differences, could introduce bias (44). The study’s strengths are first and foremost its community-based methodology and that it compares people of different ethnicities in the same location. SA and AC participants make up the majority of British first-generation migrants and, unlike in some countries, universal healthcare, free at the point of use, is available in UK. This may lessen, though not abolish disadvantages in health access (45). All examinations were conducted according to a strict approach, resulting in a comprehensive phenotyping of this older age sample.

## Conclusion

In people resident in UK, EA and SA have a higher burden of atherosclerotic plaques in carotid arteries than AC, while in contrast cIMT was higher in AC than other ethnicities. These differences were unexplained by ASCVD risk factors. The disparity between these findings and the known risks of ASCVD in these ethnic groups raises questions about the utility of carotid ultrasound as a tool to predict risk in multi-ethnic populations.

## Data availability statement

The raw data supporting the conclusions of this article will be made available by the authors, without undue reservation.

## Ethics statement

The studies involving human participants were reviewed and approved by the Ealing, Hounslow and Spelthorne, Parkside, and University College London Research Ethics Committees. The patients/participants provided their written informed consent to participate in this study.

## Author contributions

RA and ADH had full access to all the data in the study. RA performed the statistical analyses and wrote the first draft of the manuscript. All authors contributed to study design and interpretation and approved the final manuscript.
